# Epigenetic age acceleration and risk of aortic valve stenosis: a bidirectional Mendelian randomization study

**DOI:** 10.1186/s13148-024-01647-5

**Published:** 2024-03-12

**Authors:** Wanqian Pan, Qi Huang, Le Zhou, Jia Lin, Xiaojiao Du, Xiaodong Qian, Tingbo Jiang, Weixiang Chen

**Affiliations:** 1 Department of Cardiology, The First Affiliated Hospital of Soochow University, 188 Shizi Street, Suzhou, 215006 Jiangsu People’s Republic of China; 2 Department of Radiology, The First Affiliated Hospital of Soochow University, Suzhou City, 215000 Jiangsu Province People’s Republic of China

**Keywords:** Aortic valve stenosis, Bidirectional Mendelian randomization, Biological aging, Epigenetic clock, Genome-wide association study

## Abstract

**Background:**

Aortic valve stenosis (AVS) is the most prevalent cardiac valve lesion in developed countries, and pathogenesis is closely related to aging. DNA methylation-based epigenetic clock is now recognized as highly accurate predictor of the aging process and associated health outcomes. This study aimed to explore the causal relationship between epigenetic clock and AVS by conducting a bidirectional Mendelian randomization (MR) analysis.

**Methods:**

Summary genome-wide association study statistics of epigenetic clocks (HannumAge, HorvathAge, PhenoAge, and GrimAge) and AVS were obtained and assessed for significant instrumental variables from Edinburgh DataShare (*n* = 34,710) and FinnGen biobank (cases = 9870 and controls = 402,311). The causal association between epigenetic clock and AVS was evaluated using inverse variance weighted (IVW), weighted median (WM), and MR-Egger methods. Multiple analyses (heterogeneity analysis, pleiotropy analysis, and sensitivity analysis) were performed for quality control assessment.

**Results:**

The MR analysis showed that the epigenetic age acceleration of HorvathAge and PhenoAge was associated with an increased risk of AVS (HorvathAge: OR = 1.043, *P* = 0.016 by IVW, OR = 1.058, *P* = 0.018 by WM; PhenoAge: OR = 1.058, *P* = 0.005 by IVW, OR = 1.053, *P* = 0.039 by WM). Quality control assessment proved our findings were reliable and robust. However, there was a lack of evidence supporting a causal link from AVS to epigenetic aging.

**Conclusion:**

The present MR analysis unveiled a causal association between epigenetic clocks, especially HorvathAge and PhenoAge, with AVS. Further research is required to elucidate the underlying mechanisms and develop strategies for potential interventions.

**Graphical abstract:**

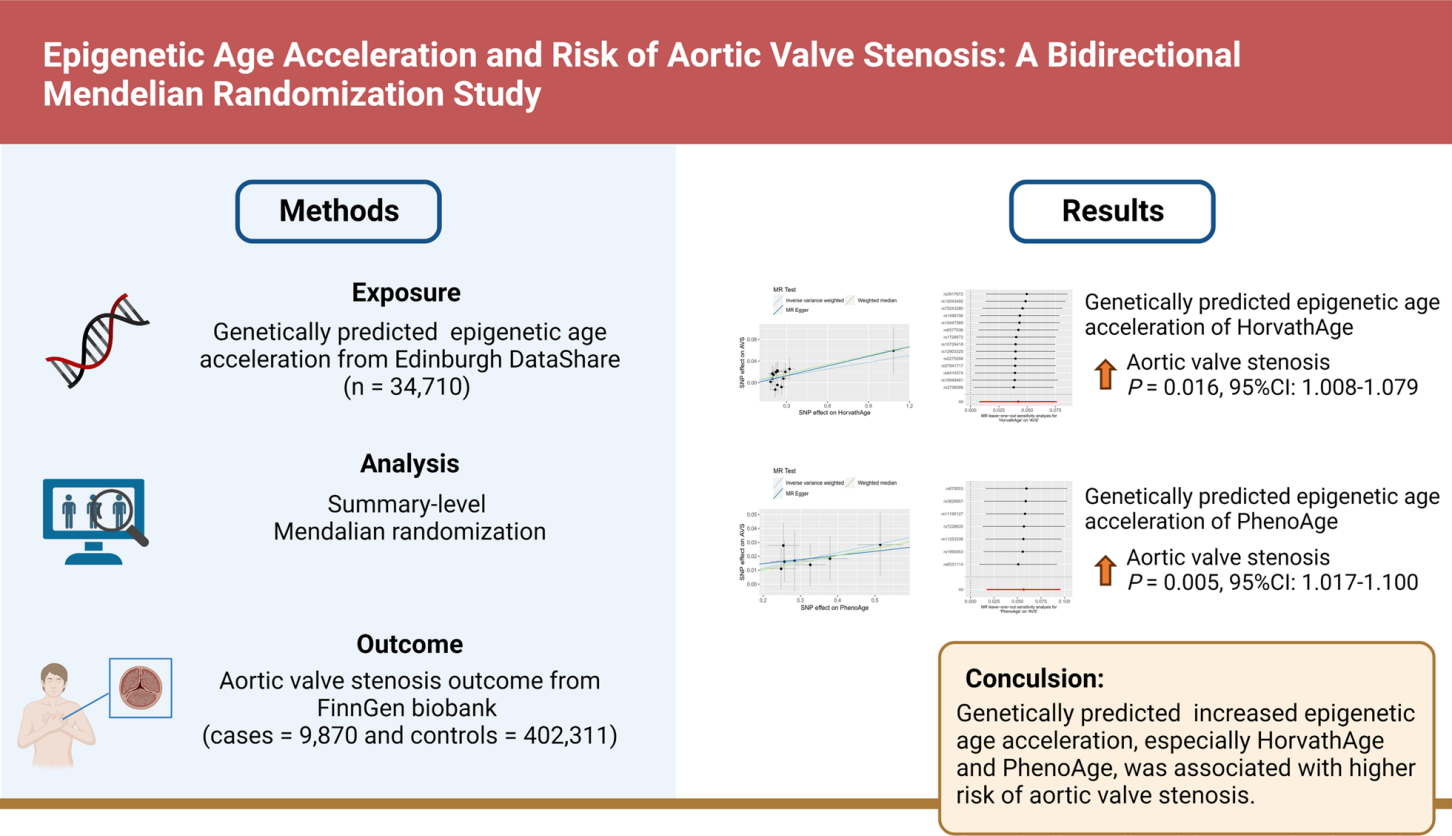

**Supplementary Information:**

The online version contains supplementary material available at 10.1186/s13148-024-01647-5.

## Introduction

Aortic valve stenosis (AVS), the most prevalent valvular heart disease, is characterized by progressive fibro-calcific remodeling and thickening of the aortic valve cusps [1, 2]. It is most often degenerative in pathogenesis, with a prevalence of only 0.02% among subjects aged 18–44 years but 2.8% in patients aged ≥ 75 years [3]. Symptomatic AVS is associated with a dismal prognosis, with a mortality rate of more than 50% at 2 years [4]. However, no pharmacotherapy has been proven to reverse aortic valve calcification effectively [5]. Most patients would eventually require surgical or transcatheter aortic valve repair or replacement [6].

The epigenetic clock is currently the best predictor of biological aging status compared to chronological age and other age-related biomarkers (e.g., telomere length) [7, 8]. Each epigenetic clock reflects biological aging profiles by measuring DNA methylation (DNAm) levels at specific cytosine-phosphate-guanine (CpG) loci. “First generation” epigenetic clocks, like HannumAge and HorvathAge, utilize DNAm levels at CpG loci closely linked to actual age for their calculations [9, 10]. “Second generation” epigenetic clocks, like PhenoAge and GrimAge, exhibit a commendable ability to forecast age-related morbidity and mortality [11, 12]. Epigenetic age acceleration (EAA) is employed to characterize individuals whose estimated physiological age exceeded their actual chronological age, which is strongly related to the development of cardiovascular diseases [13, 14]. Although AVS is associated with senescence [15], no research has been conducted to explore the relationship between the epigenetic aging acceleration and AVS.

Mendelian randomization (MR) leverages genetic variants as instrumental variables (IVs) to support the causal inference without confounding and reverse causation, with random genotype allocation mimicking randomized controlled trials [16]. In this analysis, we performed a bidirectional two-sample MR analysis to investigate the causal association between epigenetic clocks (HannumAge, HorvathAge, PhenoAge, and GrimAge) and the risk of AVS.

## Method

### Study design

**Fig. 1:**
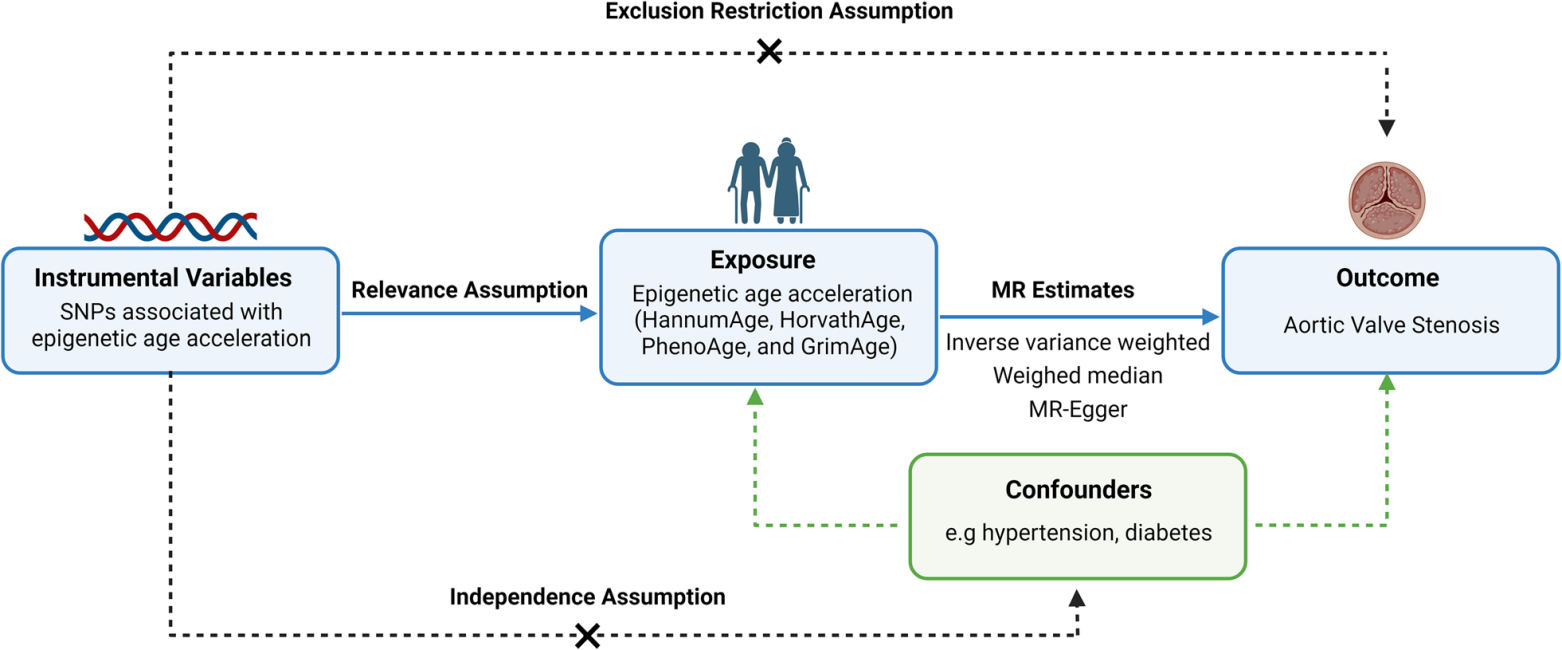
The flowchart of present study and basic assumptions of MR analysis. The objective of this two-sample bidirectional MR analysis is to investigate the causality between epigenetic age acceleration and AVS. The GWAS meta-analysis utilized in this study is from mixed-sex European cohorts. Abbreviation: AVS: aortic valve stenosis; GWAS: genome-wide association studies; MR: Mendelian randomization; SNPs: single-nucleotide polymorphisms

MR analysis should adhere to the following three key assumptions. (1) relevance assumption: IVs must be closely correlated with the exposure phenotype. (2) independence assumption: IVs should be independent of any confounder factors. (3) exclusion-restriction assumption: IVs only influence the outcome through exposure phenotype. An overview of the principle, design, and process of the present MR study is shown in Fig. 1.

### Data source

The genetic instruments for four epigenetic age measures, namely HannumAge, HorvathAge, PhenoAge, and GrimAge, were obtained from the recent genome-wide association studies (GWAS) meta-analysis based on 28 cohorts of 34,710 European ancestry participants [17]. Summary-level data on AVS were obtained from the GWAS meta-analysis of 412,181 individuals (9870 cases and 402,311 controls) of European descent conducted by FinnGen Project Database. The original data utilized in this study were approved by the ethical committee, and all participants have duly provided their consent forms.

### Selection of genetic instrumental variants

Single-nucleotide polymorphisms (SNPs) with genome-wide significant associations with exposure (*p* < 5 × 10^–8^) were selected as IVs. Because only four SNPs were selected for GrimAge, the restriction was loosened to a threshold of 5 × 10^–6^ to identify a suitable number of IVs [18]. Establishing criteria of *r*^2^ < 0.001, and kb > 10,000 to eliminate linkage disequilibrium [19]. Then, we deleted IVs surrogating confounders and outcomes to fulfill the second and third MR assumptions by querying the traits proxied of each SNP in the PhenoScannerV2 database. All outlier and palindromic SNPs were removed.

The proportion of phenotypic variations explained by tool variables (*R*^2^) and tool strength (*F*-statistics) were performed to avoid weak shifts in IVs by using the formulas: *R*^2^ = [2 × (1 − MAF) × MAF × *β*^2^]/(SE^2^ × *N*) and *F*-statistic = [(*N* − *k* − 1)/k] × [*R*^2^/(1 − *R*^2^)], where SE is the standard error, β is the effect size, MAF is the minor allele frequency for each SNP, k presents the number of SNPs, and *N* presents the sample size. *F*-statistic ≥ 10 is considered as strong genetic instrument to explain phenotypic variations [20]. Strong genetic instruments were chosen as the IVs of exposure phenotype for MR analysis.

### MR analysis

We evaluated the causal associations between epigenetic aging and AVS by using three distinct methods: inverse variance weighted (IVW) with fixed-effects model, MR-Egger, and weighted median (WM) [21]. When statistically significant heterogeneity was present, we used IVW with multiplicative random-effects model for MR analysis [22]. The IVW method combines the Wald estimates of genetically causal associations for each SNP to evaluate the impact of exposure on outcome, which operates under the assumption that all selected SNPs are valid IVs. It can provide the most accurate estimate and is employed as the principal statistical approach to evaluate the causal effect [23]. WM and MR-Egger were applied to complement the MR results. When more than 50% of the selected SNPs are used as IVs, the WM method produces a consistent estimate of the final estimate [24]. The MR-Egger method provides an estimate with adjustment for horizontal pleiotropy if any [25].

### Heterogeneity, pleiotropy, and sensitivity assessment

Cochran's *Q* statistic was performed to assess the heterogeneity among the SNPs for exposure. *Q*-statistic and *I*^2^ (%)-value could quantitatively assess the heterogeneity, which is calculated as *I*^2^ = [*Q* − (*K* − 1)]/*Q* (*K* presents the number of SNPs, *Q* is *Q*-statistic) [23]. The horizontal pleiotropy was analyzed by MR-Egger intercept and MR-PRESSO Global test methods. The proximity of the intercept to zero indicates a lower likelihood of horizontal pleiotropy [26]. Employing the leave-one-out sensitivity analysis, we examined whether individual SNPs exerted significant influence on the overall causal estimates by removing each SNP [27].

### Statistical analysis

All statistical analyses and result visualizations were implemented by using R software 4.3.1 (R Foundation for Statistical Computing, Vienna, Austria) with the "TwoSampleMR", "LDlinkR", and "forestplot" Packages.

## Results

### Causal analysis of epigenetic aging on AVS

After calculating *F*-statistics and querying the proxied traits, we screened significant correlated SNPs as strong IVs of epigenetic aging for MR analyses (HannumAge = 4, *R*^2^ = 0.306%, *F* = 26.587; HorvathAge = 14, *R*^2^ = 0.808%,* F* = 20.189; PhenoAge = 7, *R*^2^ = 0.374%, *F* = 18.632; GrimAge = 3, *R*^2^ = 0.102%, *F* = 11.755) (Additional file 1: Table S1).

**Fig. 2:**
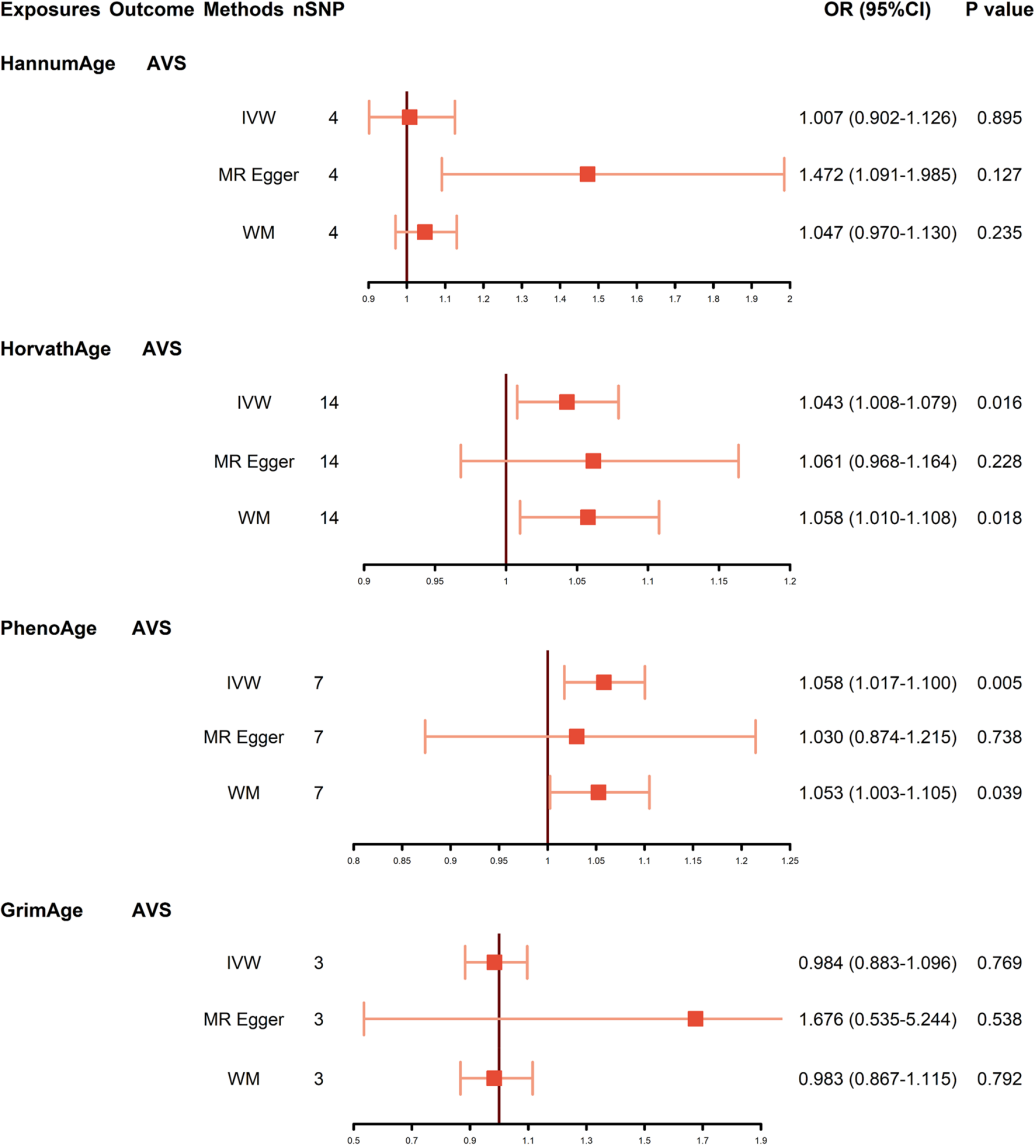
Causal estimates from genetically predicted epigenetic age to AVS. Visualization of the results of three MR analysis methods. Abbreviations: AVS: aortic valve stenosis; CI: confidence interval; IVW: inverse variance weighted; MR: Mendelian randomization; OR: odds ratio; SNP: single-nucleotide polymorphism; WM: weighted median

**Fig. 3:**
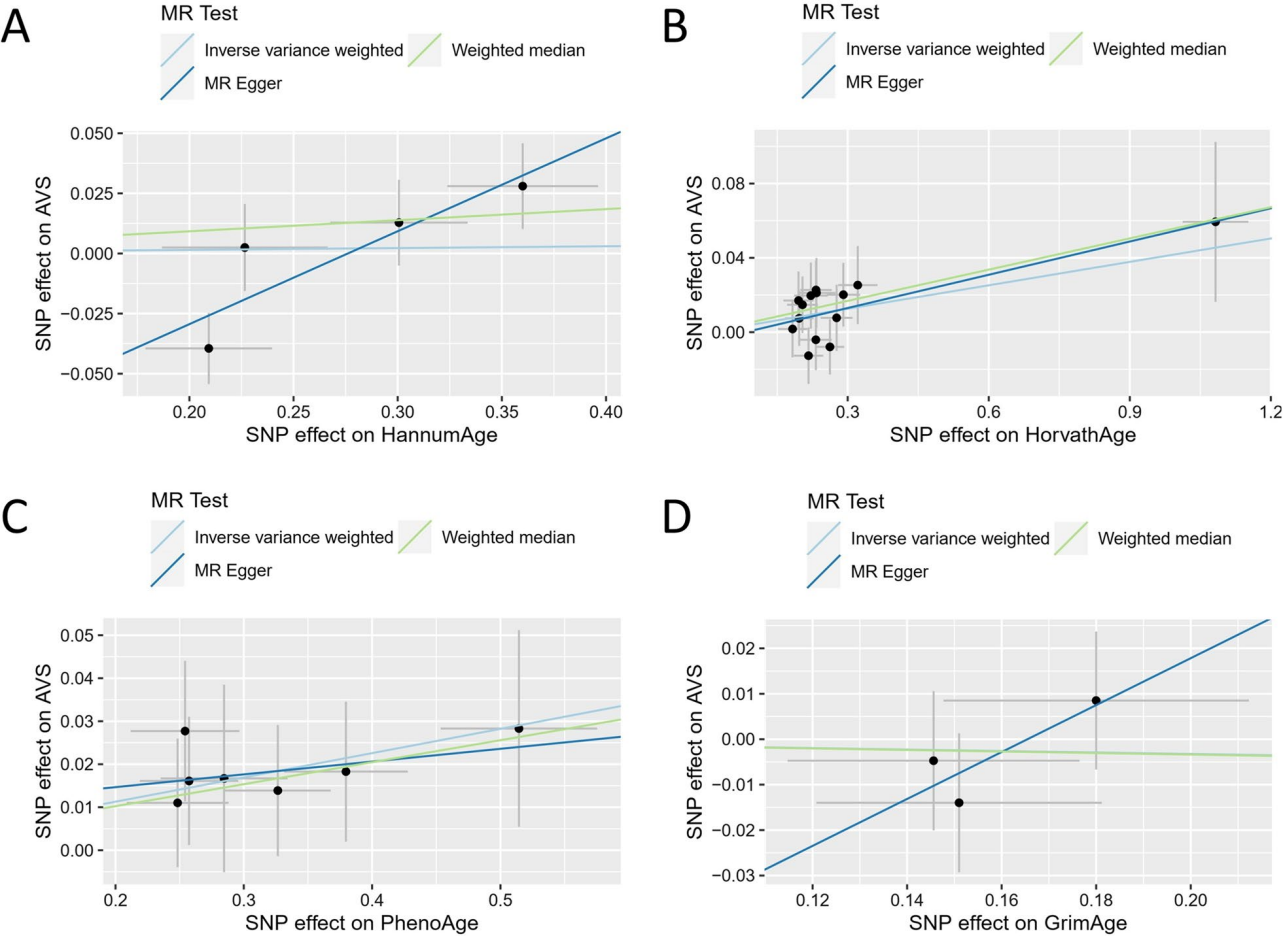
Scatter plots of epigenetic age and AVS. HannumAge (**A**), HorvathAge (**B**), PhenoAge (**C**), and GrimAge (**D**) as exposure and AVS as outcome. Abbreviations: AVS: aortic valve stenosis; MR: Mendelian randomization; SNP: single-nucleotide polymorphism

As plotted in Fig. 2, genetically predicted HorvathAge was significantly associated with AVS (95% CI 1.008–1.079, *P* = 0.016 by IVW; 95% CI 1.010–1.108, *P* = 0.018 by WM). MR analyses also indicated a causal relationship between PhenoAge and AVS (95% CI 1.017–1.100, *P* = 0.005 by IVW; 95% CI 1.003–1.105, *P* = 0.039 by WM). However, we did not observe the causal association between other epigenetic clocks and the odds of AVS (HannumAge: 95% CI 0.902–1.126, *P* = 0.895; GrimAge: 95% CI 0.883–1.096, *P* = 0.769). Figure 3 exhibits the scatter plots of the three methods. The trend lines indicated that genetically predicted increased HorvathAge and PhenoAge were related to a higher risk of AVS. The forest plots of individual SNP effect of epigenetic aging on AVS are shown in Additional file 1: Fig. S1.

### Quality control assessment for forward MR analysis

**Fig. 4:**
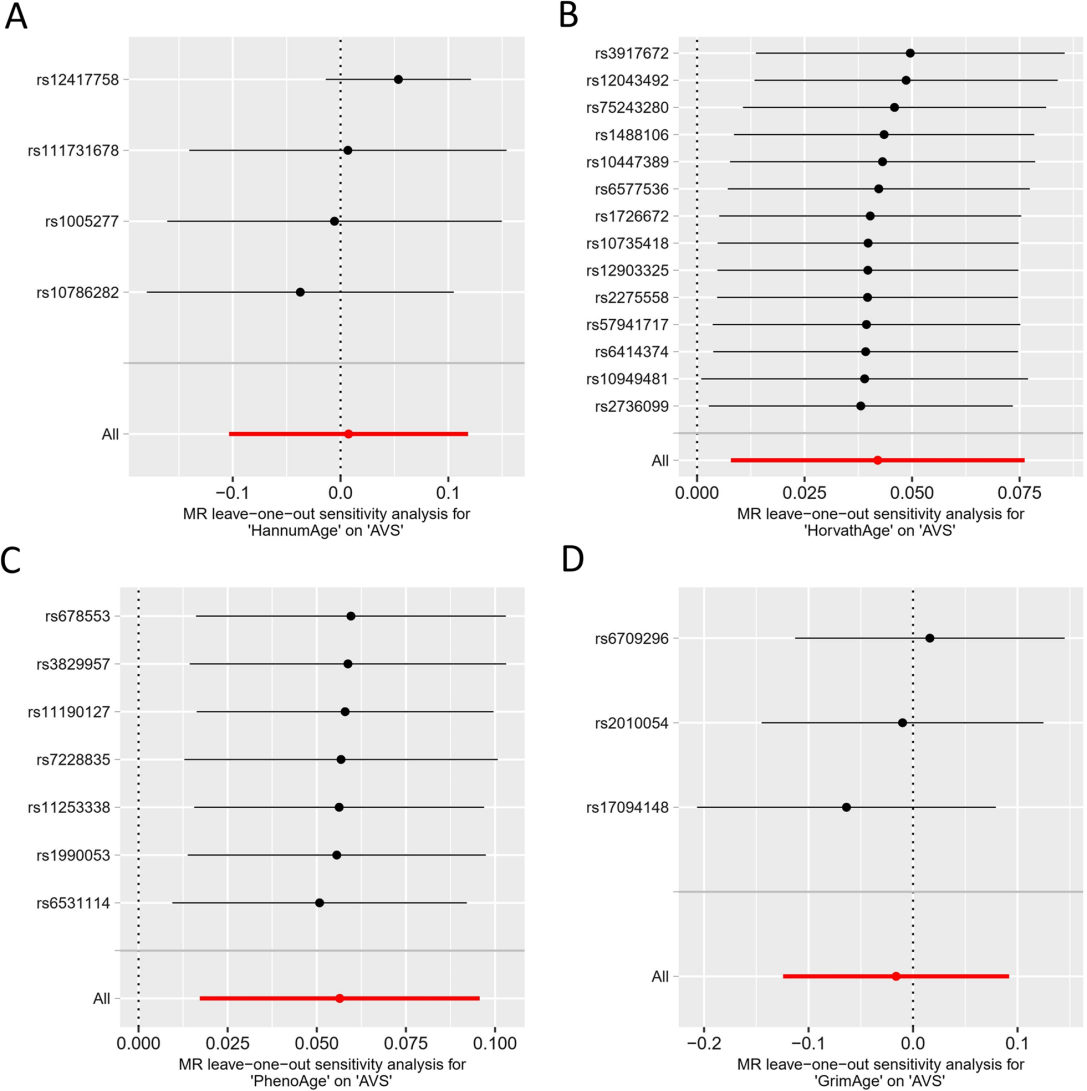
Leave-one-out analysis of epigenetic age and AVS. Sensitivity analysis for HannumAge (**A**), HorvathAge (**B**), PhenoAge (**C**), and GrimAge (**D**) on AVS. Abbreviations: AVS: aortic valve stenosis; MR: Mendelian randomization

By measuring Cochran’s *Q* test, heterogeneity was detected in HannumAge (*P* = 0.018, *Q* = 10.064, *I*^2^ = 70.1%). As a result, we performed IVW with multiplicative random-effects model for MR analysis. No significant heterogeneity between other epigenetic clocks and AVS (HorvathAge: *P* = 0.897, *Q* = 7.092, *I*^2^ = 0%; PhenoAge: *P* = 0.991, *Q* = 0.850, *I*^2^ = 0%; GrimAge: *P* = 0.560, *Q* = 1.160, *I*^2^ = 0%). Table 1 exhibits the results of the MR-Egger intercept and MR-PRESSO Global test, indicating the absence of horizontal pleiotropy among all analyses. After one SNP was removed at a time and the remaining SNPs were analyzed, no significant changes in overall effect estimates were observed (Fig. 4). These findings suggested that our MR results had significant confidence with good robustness and steadiness.

**Table 1:** Heterogeneity, pleiotropy, and tool strength of MR analyses

Exposure	Outcome	Cochran’s *Q* statistic			Egger intercept		Global test	*F*-statistic
		*P* value	*Q*	*I*^2^ (%)	*P *value	Intercept	*P* value	
GrimAge	AVS	0.560	1.160	0.0	0.527	− 0.085	–	11.755
HannumAge	AVS	0.018	10.046	70.1	0.126	− 0.107	0.077	26.587
HorvathAge	AVS	0.897	7.092	0.0	0.693	− 0.005	0.904	20.189
PhenoAge	AVS	0.991	0.850	0.0	0.757	0.009	0.993	18.632
AVS	GrimAge	0.636	13.494	0.0	0.684	0.010	0.667	20.565
AVS	HannumAge	0.157	20.398	26.5	0.542	0.018	0.186	20.958
AVS	HorvathAge	0.339	17.739	9.8	0.879	− 0.004	0.346	20.565
AVS	PhenoAge	0.870	9.930	0.0	0.830	− 0.007	0.887	20.565

### Causal analysis of AVS on epigenetic aging

**Fig. 5:**
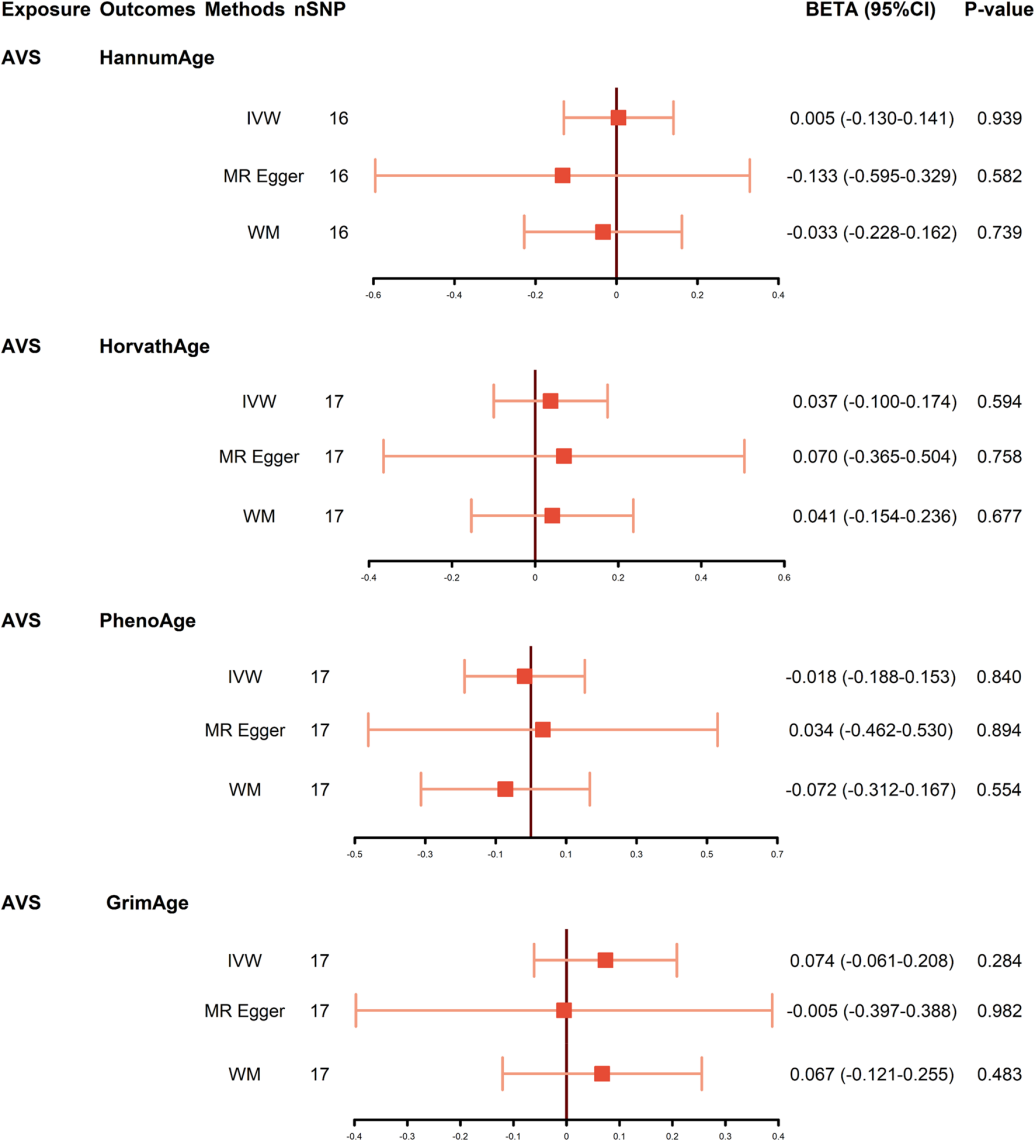
Causal estimates from genetically predicted AVS to epigenetic age. Visualization of the results of three MR analysis methods. Abbreviations: AVS: aortic valve stenosis; CI: confidence interval; IVW: inverse variance weighted; MR: Mendelian randomization; OR: odds ratio; SNP: single-nucleotide polymorphism; WM: weighted median

Then, we performed MR analysis with AVS as exposure to explore the possible reverse causality on epigenetic aging. As shown in Fig. 5, genetic predicted AVS was not associated with any epigenetic aging-related traits (HannumAge: *P* = 0.939, 95% CI − 0.130 to 0.141; HorvathAge: *P* = 0.594, 95% CI − 0.100 to 0.174; PhenoAge: *P* = 0.840, 95% CI − 0.188 to 0.153; GrimAge: *P* = 0.284, 95% CI − 0.061 to 0.208). Neither heterogeneity nor pleiotropy was detected in the reverse directional MR analysis (Table 1). The scatter plots and leave-one-out of the genetic variance are presented in Additional file 1: Figs. S2-3.

## Discussion

To the best of our knowledge, this is the first MR analysis to explore the bidirectional causal association between epigenetic clock and AVS. In the present research, we found that increased genetically predicted HorvathAge and PhenoAge were related to a higher risk of AVS. Conversely, the result did not support the causal relationship of AVS on any epigenetic clocks. It is suggested that EAA of HorvathAge and PhenoAge is the risk factor for the prevalence of AVS.

Degenerative lesion is the most common etiology of AVS [28]. Age-related cellular and stress-induced senescence, accompanied by subsequent active processes, constitute crucial elements in the pathomechanism of AVS [29]. Age-related senescent cells release cytokines, chemokines, and matrix metalloproteinases, known as senescence-associated secretory phenotypes. It brings about increased collagen content and leaflet stiffness with extracellular matrix remodeling and structural changes in the valvular tissue [30, 31]. In addition, DNA damage can be triggered by cellular stressors, like excessive mechanical stress, metabolic stress, and oxidative stress, referred to as stress-induced premature cellular senescence [32, 33]. Constant hemodynamic stress-induced endothelial denudation is repaired by circulating endothelially progenitor cells. However, aging-induced reduction in the number of circulating endothelial progenitor cells impedes the clearance of senescent endothelial cells, leading to activate reactive oxygen species, inflammatory responses, and activating the lipid infiltration pathway [34]. Sirtunin1, an NAD (+)-dependent deacetylase, exerts anti-aging effects by controlling mitochondrial biogenesis and oxidative stress [35]. Sophie Carter et.al found that the expression of Sirtunin1 was reduced in explanted valves from AVS patients [36]. Targeted modulation of Sirtunin1 is a potential therapy for AVS [37].

Previous studies have revealed a correlation between DNA methylation and AVS. Based on the Illumina 450 k Beadchip and enzyme-linked immunosorbent assay methods, Nwachukwu et al. identified more than 6,000 differently methylated sites between normal and aortic stenotic tissue. The increased DNA methylation of DNA methyltransferase 3 beta activated the osteogenic pathways in valves [38]. Fayez Hadji et.al reported the dysregulation of DNA methylation in the promoter of H19 by performing multidimensional genomic profiling in human calcific aortic valves. The overexpression of H19 promoted the osteogenic program through impeding NOTCH1 transcription [39]. Takahito Nasu et.al found that the DNA methylation in the region encoding tribbles homolog 1 was lower in the AVS group than in the controls by analyzing epigenome-wide association study of peripheral blood mononuclear cells, which may be the result of hemodynamic overload [40]. In addition, the osteogenic transition of valve interstitial cells was promoted by DNA methylation-mediated downregulation of phospholipid phosphatase 3 [41].

Because there is significant heterogeneity in health aging [42], we need better predictors to understand and measure senescence than chronological age. The epigenetic clock and telomere length are regarded as the most compelling predictors of biological age [8]. Based on the Southern blot hybridization and quantitative polymerase chain reaction, David J Kurz et.al revealed that AVS was associated with shorter telomere length in the elderly [43]. Experiments carried out by Ilona Saraieva also confirmed the result [44]. However, the predictive ability of telomere length is low [8]. Although studies have reported the relationship between the epigenetic clock and cardiovascular diseases, there is no research analyzing the influence of epigenetic aging on the pathogenesis of AVS. A German case-cohort study reported an increased risk of cardiovascular death associated with EAA of HorvathAge [45], contrary to the findings of a study in the Melbourne Collaborative Cohort [46]. What’s more, EAA of PhenoAA, rather than HorvathAA, was related to an increase in the hazard of cardiovascular death in the US Normative Aging Study [47]. These contradictory results may result from the small number of cases and short follow-up time.

In the present research, we explored the causal association between epigenetic clock and AVS by MR analyses. Using the fundamental concept of the random allocation of alleles during zygote formation, MR analysis can establish dependable causal inferences overcoming confounding and reverse causality biases [48]. To mitigate potential bias arising from group stratification, only GWAS data derived from individuals of European descent were utilized in this study. Moreover, our dataset was obtained from the Edinburgh DataShare and the FinnGen Project Database, ensuring no overlap in samples. Quality control assessment demonstrated that our results were reliable and robust. This study expanded the knowledge of risk factors for AVS that EAA of HorvathAge and PhenoAge was associated with higher odds of AVS. Epigenetic clocks may become a surveillance indicator for clinicians and preventive medicine practitioners to assess the risk of developing AVS. Decelerating biological aging has emerged as a novel research focus in the prevention of AVS.

Our study also has inevitable limitations. First, the pooled GWAS data utilized in our study originated from populations of European ancestry, raising concerns about the generalizability of our findings to other ethnic groups. Second, our study could not perform a stratified analysis of the progression and severity of AVS due to the lack of publicly available dataset. Third, the ORs in our findings are low, necessitating cautious interpretation of the results. Finally, since epigenetic aging is essentially related to environmental exposures rather than genetic factors, this highlights the limitations of applying MR in this context.

## Conclusion

Our findings suggested the potential causal relationship of accelerated epigenetic clocks, especially HorvathAge and PhenoAge, to the risk of AVS. Slowing down biological aging has emerged as a new research direction in curbing AVS.

### Supplementary Information

**Additional file 1. Table S1.** Summary statistics of the EAA genetic instrumental variables. **Table S2.** Summary statistics of the AVS genetic instrumental variables in reverse MR analysis. **Fig S1.** The forest plots of EAA on AVS. **Fig S2.** The scatter plots of AVS on EAA. **Fig S3.** The leave-one-out plots of AVS on EAA. **Fig S4.** The forest plots of AVS on EAA.

## Data Availability

No original data were generated in the present study. The datasets mentioned in this article are publicly available in the Edinburg DataShare (https://datashare.ed.ac.uk/handle/10283/3645) for GWAS of epigenetic clocks and FinnGen biobank (phenocode: I9_CAVS_OPERATED, https://www.finngen.fi/en/access_results) for GWAS of AVS.

## Abbreviations

AVS: Aortic valve stenosis
EAA: Epigenetic age acceleration
GWAS: Genome-wide association studies
IVW: Inverse variance weighted
MR: Mendelian randomization
OR: Odds ratio
SNPs: Single-nucleotide polymorphisms
WM: Weighted median
95% CI: 95% Confidence interval
