# Supplementary material for: Epigenetic age acceleration and risk of aortic valve stenosis: a bidirectional Mendelian randomization study
Source: Clin Epigenetics. 2024 Mar 12;16:41. doi: 10.1186/s13148-024-01647-5 (PMC10936111; doi:10.1186/s13148-024-01647-5)
Supplement: Supplementary file 1 — Additional file 1. Table S1. Summary statistics of the EAA genetic instrumental variables. Table S2. Summary statistics of the AVS genetic instrumental variables in reverse MR analysis. Fig S1. The forest plots of EAA on AVS. Fig S2. The scatter plots of AVS on EAA. Fig S3. The leave-one-out plots of AVS on EAA. Fig S4. The forest plots of AVS on EAA. [file 13148_2024_1647_MOESM1_ESM.docx]

***Supplementary Materials***

**Epigenetic Age Acceleration and Risk of Aortic Valve Stenosis: A Bidirectional Mendelian Randomization Study**

Wanqian Pan, Qi Huang, Le Zhou Jia Lin, Xiaojiao Du, Xiaodong Qian, Tingbo Jiang, Weixiang Chen

**Contents**

**Table S1.** Summary statistics of the EAA genetic instrumental variables.

**Table S2.** Summary statistics of the AVS genetic instrumental variables in reverse MR analysis.

**Figure S1.** The forest plots of EAA on AVS.

**Figure S2.** The scatter plots of AVS on EAA.

**Figure S3.** The leave-one-out plots of AVS on EAA.

**Figure S4.** The forest plots of AVS on EAA.

**Table S1.** Summary statistics of the EAA genetic instrumental variables.

| Exposure | SNP | A1 | A2 | Beta | Eaf | Se | *P*-value | R2 | F-statistic |
| --- | --- | --- | --- | --- | --- | --- | --- | --- | --- |
| HannumAge | rs1005277 | A | C | 0.30 | 0.30 | 0.03 | 8.92E-20 | 0.10% | 35.41 |
|  | rs10786282 | A | G | -0.36 | 0.21 | 0.04 | 2.61E-23 | 0.10% | 36.36 |
|  | rs111731678 | A | T | -0.23 | 0.19 | 0.04 | 1.26E-08 | 0.03% | 10.84 |
|  | rs12417758 | T | C | -0.21 | 0.55 | 0.03 | 6.22E-12 | 0.07% | 23.52 |
| HorvathAge | rs10447389 | A | G | -0.28 | 0.27 | 0.03 | 4.42E-16 | 0.08% | 27.12 |
|  | rs10735418 | T | C | 0.20 | 0.63 | 0.03 | 1.14E-09 | 0.05% | 18.47 |
|  | rs10949481 | A | T | 1.08 | 0.95 | 0.07 | 4.56E-54 | 0.04% | 14.09 |
|  | rs12043492 | T | C | 0.22 | 0.42 | 0.03 | 4.35E-12 | 0.07% | 23.66 |
|  | rs12903325 | T | G | -0.22 | 0.76 | 0.04 | 5.48E-10 | 0.04% | 13.32 |
|  | rs1488106 | T | C | 0.18 | 0.37 | 0.03 | 4.25E-09 | 0.04% | 15.46 |
|  | rs1726672 | T | C | -0.20 | 0.30 | 0.03 | 6.05E-10 | 0.05% | 16.51 |
|  | rs2275558 | A | G | -0.23 | 0.22 | 0.04 | 6.44E-09 | 0.03% | 10.88 |
|  | rs2736099 | A | G | 0.23 | 0.35 | 0.03 | 3.47E-12 | 0.07% | 22.59 |
|  | rs3917672 | A | G | -0.26 | 0.49 | 0.03 | 5.95E-18 | 0.11% | 36.69 |
|  | rs57941717 | T | G | 0.29 | 0.25 | 0.04 | 3.71E-16 | 0.07% | 24.73 |
|  | rs6414374 | A | G | 0.32 | 0.16 | 0.04 | 1.42E-14 | 0.05% | 16.82 |
|  | rs6577536 | A | G | 0.20 | 0.48 | 0.03 | 6.73E-11 | 0.06% | 20.46 |
|  | rs75243280 | T | C | -0.23 | 0.67 | 0.03 | 7.37E-12 | 0.06% | 19.84 |
| PhenoAge | rs11190127 | A | C | 0.25 | 0.38 | 0.04 | 3.83E-10 | 0.05% | 17.66 |
|  | rs11253338 | T | C | 0.28 | 0.18 | 0.05 | 8.49E-09 | 0.03% | 10.09 |
|  | rs1990053 | A | G | 0.26 | 0.42 | 0.04 | 2.05E-11 | 0.06% | 22.28 |
|  | rs3829957 | T | C | -0.38 | 0.20 | 0.05 | 3.51E-15 | 0.06% | 19.25 |
|  | rs6531114 | T | C | -0.25 | 0.26 | 0.04 | 2.07E-09 | 0.05% | 15.86 |
|  | rs678553 | T | C | 0.33 | 0.69 | 0.04 | 2.27E-15 | 0.08% | 28.14 |
|  | rs7228835 | C | G | -0.51 | 0.12 | 0.06 | 3.30E-17 | 0.05% | 16.76 |
| GrimAge | rs17094148 | A | G | -0.18 | 0.71 | 0.03 | 2.55E-08 | 0.04% | 12.6 |
|  | rs2010054 | A | G | -0.15 | 0.40 | 0.03 | 2.51E-06 | 0.03% | 10.81 |
|  | rs6709296 | T | G | 0.15 | 0.42 | 0.03 | 5.57E-07 | 0.03% | 11.83 |

**Table S2**. Summary statistics of the AVS genetic instrumental variables in reverse MR analysis.

| SNP | A1 | A2 | Beta | Eaf | Se | P-value | R2 | F-statistic |
| --- | --- | --- | --- | --- | --- | --- | --- | --- |
| rs10455872 | A | G | 0.37 | 0.05 | 0.03 | 5.66E-31 | 6.16E-05 | 25.41 |
| rs10770612 | A | G | -0.12 | 0.24 | 0.02 | 8.72E-11 | 3.17E-05 | 13.05 |
| rs17550940 | A | C | 0.11 | 0.37 | 0.02 | 4.73E-13 | 5.26E-05 | 21.67 |
| rs1800797 | A | G | -0.12 | 0.47 | 0.01 | 1.99E-16 | 8.20E-05 | 33.81 |
| rs1819631 | G | T | -0.08 | 0.59 | 0.01 | 2.68E-08 | 3.49E-05 | 14.40 |
| rs2246363 | G | A | 0.14 | 0.76 | 0.02 | 4.14E-15 | 5.41E-05 | 22.32 |
| rs309306 | C | T | -0.10 | 0.44 | 0.01 | 2.90E-12 | 5.74E-05 | 23.66 |
| rs3129945 | G | A | 0.11 | 0.25 | 0.02 | 4.07E-10 | 2.70E-05 | 11.12 |
| rs4129225 | C | T | -0.08 | 0.37 | 0.02 | 3.91E-08 | 3.03E-05 | 12.51 |
| rs4520715 | C | T | 0.10 | 0.25 | 0.02 | 1.15E-08 | 2.45E-05 | 10.10 |
| rs528789 | T | A | 0.10 | 0.40 | 0.02 | 8.12E-11 | 4.97E-05 | 20.49 |
| rs62012629* | C | A | 0.10 | 0.37 | 0.02 | 1.35E-10 | 3.46E-05 | 14.25 |
| rs6702619 | T | G | 0.16 | 0.48 | 0.01 | 3.66E-26 | 1.36E-04 | 55.93 |
| rs7202596 | G | T | 0.10 | 0.57 | 0.02 | 5.24E-11 | 5.05E-05 | 20.83 |
| rs78707515 | G | A | 0.10 | 0.22 | 0.02 | 4.00E-08 | 2.57E-05 | 10.59 |
| rs938087 | T | C | -0.09 | 0.50 | 0.01 | 4.10E-09 | 4.19E-05 | 17.28 |
| rs99780 | C | T | -0.10 | 0.41 | 0.02 | 5.01E-12 | 5.32E-05 | 21.94 |

*When performing MR analysis for AVS on HannumAge, rs62012629 was removed due to being detected by the MR-PRESSO as an outlier.


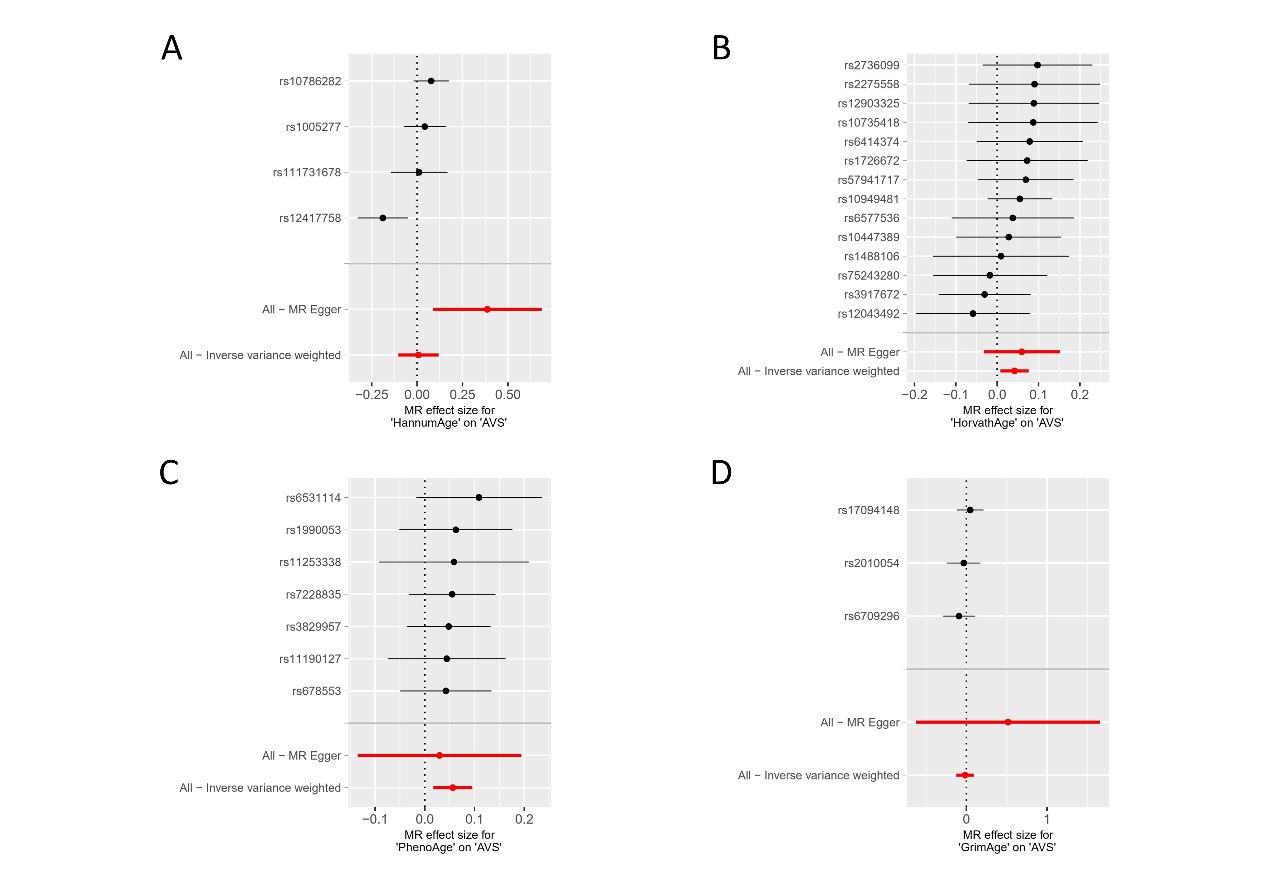


**Figure S1.** The forest plots of EAA on AVS.


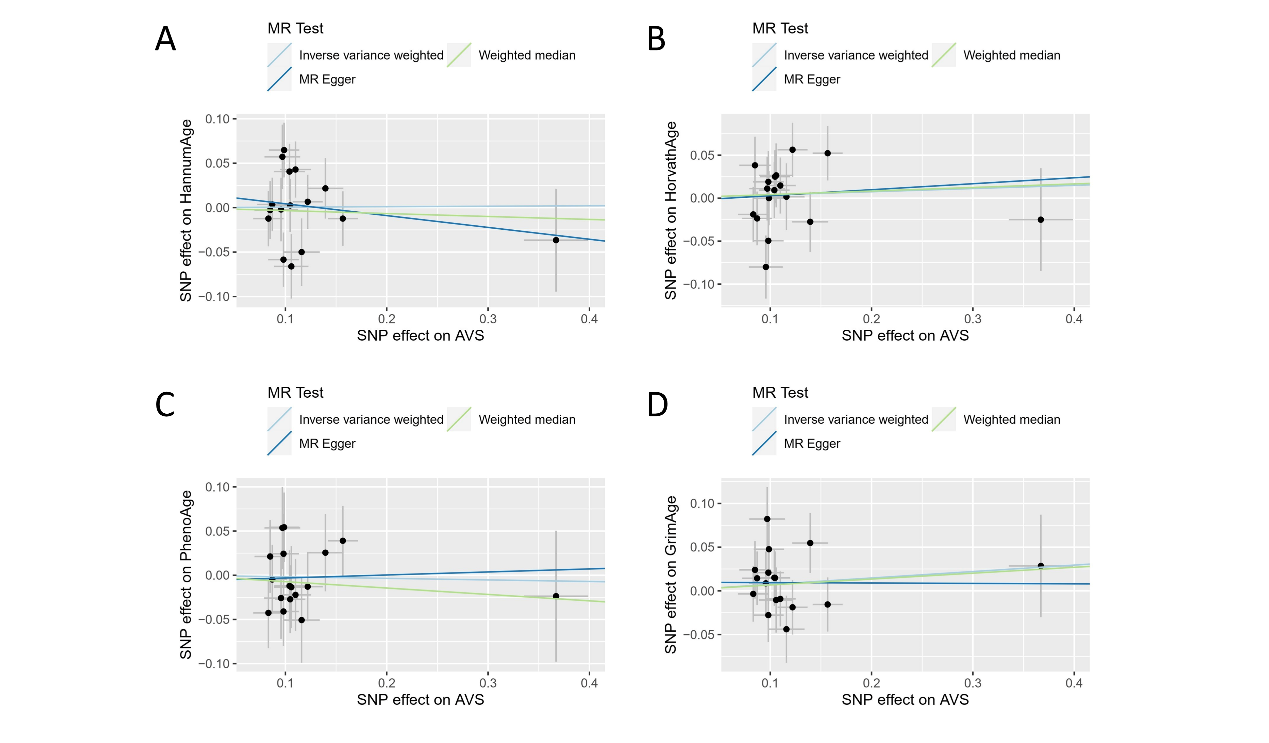


**Figure S2.** The scatter plots of AVS on EAA.


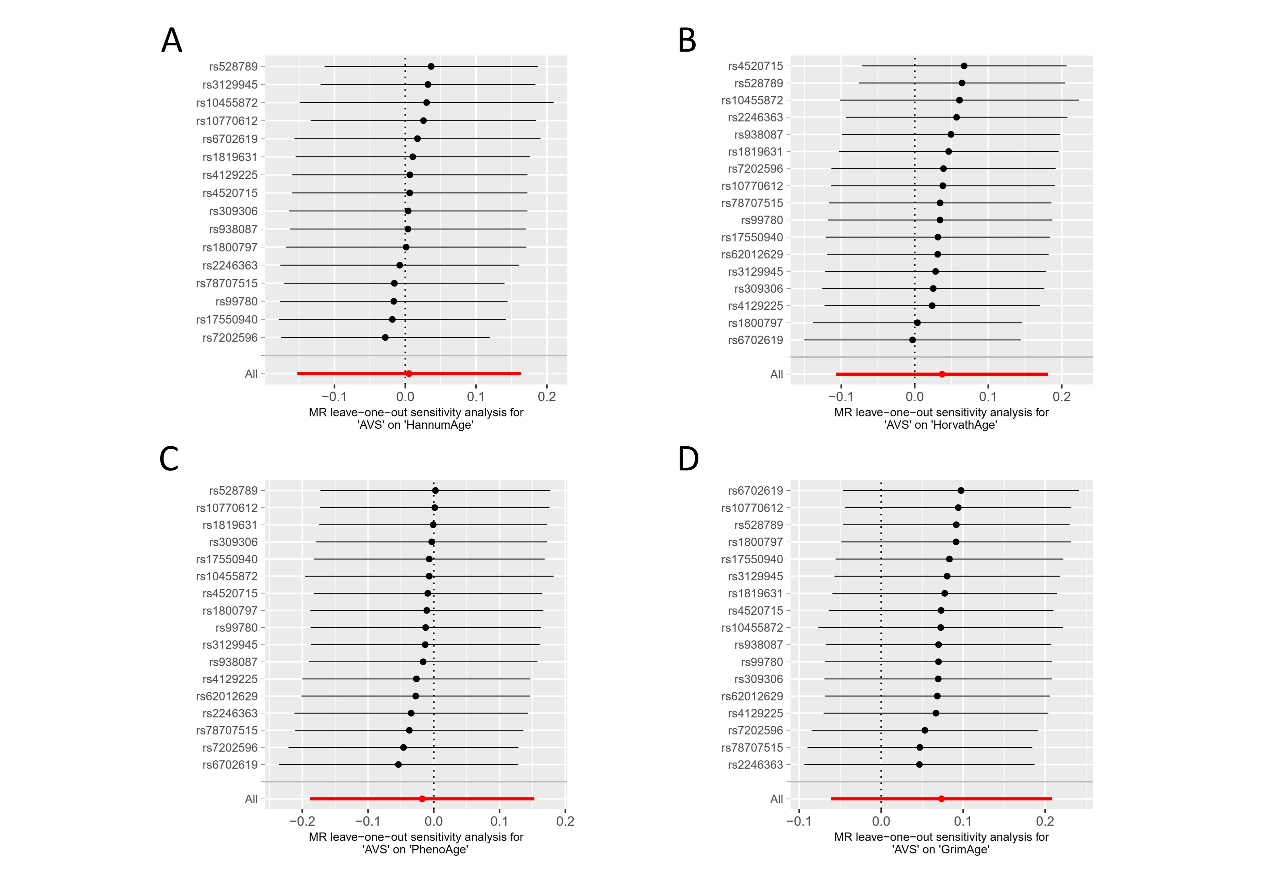


**Figure S3.** The leave-one-out plots of AVS on EAA.


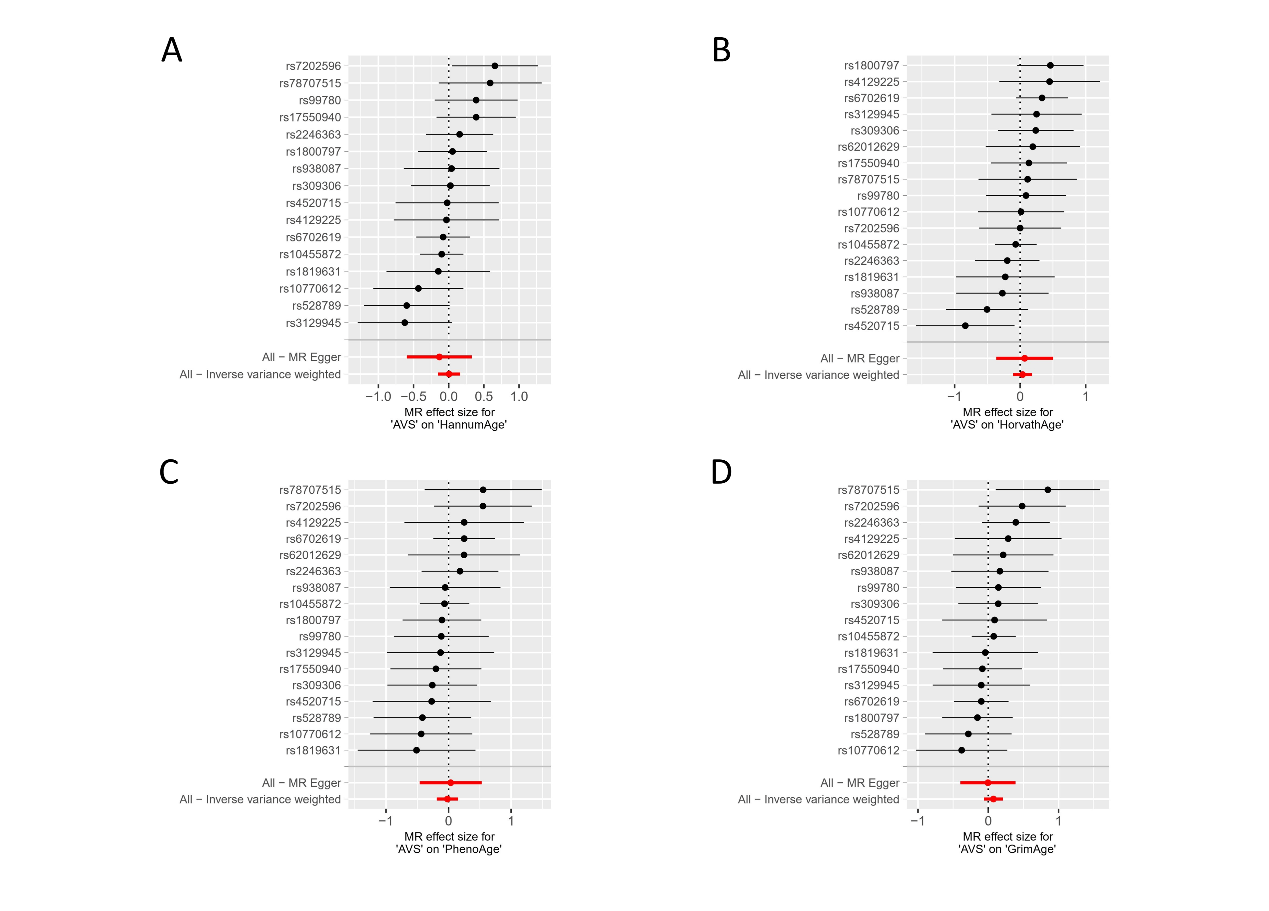
**Figure S4.** The forest plots of AVS on EAA.
